# SARS-CoV-2 infection in patients with inflammatory bowel disease: comparison between the first and second pandemic waves

**DOI:** 10.1186/s12876-023-02841-0

**Published:** 2023-07-05

**Authors:** Cristina Bezzio, Marta Vernero, Stefania Costa, Alessandro Armuzzi, Gionata Fiorino, Sandro Ardizzone, Jenny Roselli, Sonia Carparelli, Ambrogio Orlando, Flavio Andrea Caprioli, Fabiana Castiglione, Chiara Viganò, Davide G. Ribaldone, Fabiana Zingone, Rita Monterubbianesi, Nicola Imperatore, Stefano Festa, Marco Daperno, Ludovica Scucchi, Antonio Ferronato, Luca Pastorelli, Eleonora Alimenti, Paola Balestrieri, Chiara Ricci, Maria Cappello, Carla Felice, Francesca Coppini, Patrizia Alvisi, Imma Di Luna, Viviana Gerardi, Angela Variola, Silvia Mazzuoli, Marco Vincenzo Lenti, Simone Saibeni, the Italian Group for the Study of Inflammatory Bowel Disease (IG-IBD)’s COVID-19 Study Group, Daniela Pugliese, Federica Furfaro, Giovanni Maconi, Monica Milla, Fabrizio Bossa, Alessandra Giuliano, Nicole Piazza, Gianpiero Manes, Alessandro Sartini, Andrea Buda, Federica Micheli, Valeria Ciardo, Giovanni Casella, Angelo Viscido, Giorgia Bodini, Valentina Casini, Alessandra Soriano, Arnaldo Amato, Laurino Grossi, Sara Onali, Matteo Rottoli, Rocco Spagnuolo, Stefania Baroni, Claudio Cortelezzi, Monia Baldoni, Marta Vernero, Franco Scaldaferri, Giovanni Maconi, Alessia Guarino, Andrea Palermo, Renata D’Incà, Maria Lia Scribano, Livia Biancone, Lucio Carrozza, Marta Ascolani, Francesco Costa, Antonio Di Sabatino, Irene Zammarchi, Matteo Gottin, Francesco Simone Conforti

**Affiliations:** 1 Gastroenterology Unit, Rho Hospital, ASST Rhodense, Corso Europa 250, 20017 Rho, MI Italy; 2 Department of Medical Sciences, University of Turin, Turin, Italy; 3 UO Gastroenterology, ASST Milano Ovest, Legnano (MI), Legnano, Italy; 4 IBD Unit, IRCCS Humanitas Research Hospital, Rozzano, MI Italy; 5 Department of Biomedical Sciences, Humanitas University, Pieve Emanuele, MI Italy; 6 Gastroenterology and Endoscopy Unit, IRCCS Ospedale San Raffaele and University Vita-Salute San Raffaele, Milan, Italy; 7 Gastroenterology Unit, ASST Fatebenefratelli Sacco, Milan, Italy; 8 Department of Biomedical and Clinical Sciences, University of Milan, Milan, Italy; 9 Gastroenterology Department, IBD Referral Center, Azienda Ospedaliero-Universitaria Careggi, Florence, Italy; 10 Gastroenterology and Endoscopy Unit, Fondazione IRCCS Casa Sollievo Della Sofferenza, San Giovanni Rotondo, Italy; 11 IBD Unit, Villa Sofia Cervello Hospital, Palermo, Italy; 12 Gastroenterology and Endoscopy Unit, Fondazione IRCCS Ca’ Granda Ospedale Maggiore Policlinico, Milan, Italy; 13 Department of Pathophysiology and Transplantation, Università Degli Studi Di Milano, Milan, Italy; 14 Gastroenterology Unit, Department of Clinical Medicine and Surgery, University Federico II of Naples, Naples, Italy; 15 European Reference Network on Hepatological Diseases (ERN RARE-LIVER), Fondazione IRCCS San Gerardo dei Tintori, Monza, Italy; 16 Division of Gastroenterology, Department of Medical Sciences, Università Di Torino, Turin, Italy; 17 Department of Surgical, Oncological and Gastroenterological Sciences - DISCOG, University Hospital, Padua, Italy; 18 Gastroenterology and Endoscopy Unit, Azienda Ospedaliera San Camillo Forlanini, Rome, Italy; 19 Gastroenterology and Endoscopy Unit, P.O. Santa Maria delle Grazie, Pozzuoli, Naples, Italy; 20 IBD Unit, San Filippo Neri Hospital, Rome, Italy; 21 Gastroenterology Unit, Mauriziano Hospital, Turin, Italy; 22 Department of Systems Medicine, University Tor Vergata, Rome, Italy; 23 UOSD Endoscopia Digestiva, Ospedale Alto Vicentino, AULSS 7 Pedemontana, Santorso, VI Italy; 24 Gastroenterology and Liver Unit, School of Medicine at Ospedale San Paolo, Department of Health Sciences, ASST Santi Paolo E Carlo, University of Milan, Milan, Italy; 25 Gastroenterology and Endoscopy Unit, Policlinico Campus Bio Medico, Rome, Italy; 26 Gastroenterology Unit, Clinical and Experimental Sciences Department, Spedali Civili Hospital, University of Brescia, Brescia, Italy; 27 Gastroenterology and Hepatology Section, Department of Health Promotion, Mother and Child Care, Internal Medicine and Medical Specialties, University of Palermo, Palermo, Italy; 28 Medicine 1 Unit, Ca’ Foncello University Hospital, Department of Medicine (DIMED), University of Padova, Padova, Italy; 29 Department of Translational Research and New Technologies in Medicine and Surgery, University of Pisa, Pisa, Italy; 30 Pediatric Gastroenterology Unit, Maggiore Hospital, Bologna, Italy; 31 Medicine, Gastroenterology and Digestive Endoscopy Department, Fondazione Poliambulanza, Brescia, Italy; 32 IBD Unit, IRCCS Sacro Cuore Don Calabria, Negrar, VR Italy; 33 Gastroenterology and Artificial Nutrition Department, Ospedale Monsignor Raffaele Dimiccoli, Barletta, BT Italy; 34 Department of Internal Medicine and Medical Therapeutics, University of Pavia, Pavia, Italy; First Department of Internal Medicine, San Matteo Hospital Foundation, Pavia, Italy

**Keywords:** SARS-CoV-2, COVID-19, Outcome, Pandemic, Inflammatory bowel disease

## Abstract

**Background:**

In Italy, the incidence of SARS-CoV-2 infection peaked in April and November 2020, defining two pandemic waves of coronavirus disease 2019 (COVID-19). This study compared the characteristics and outcomes of patients with inflammatory bowel disease (IBD) and SARS-CoV-2 infections between pandemic waves.

**Methods:**

Observational longitudinal study of IBD patients with SARS-CoV-2 infection. Patients with established diagnoses of IBD and of SARS-CoV-2 infection were consecutively enrolled in two periods: (i) first wave, from 1 March 2020 to 31 May 2020; and (ii) second wave, from 15 September to 15 December 2020.

**Results:**

We enrolled 937 IBD patients (219 in the first wave, 718 in the second wave). Patients of the first wave were older (mean ± SD: 46.3 ± 16.2 vs. 44.1 ± 15.4 years, *p* = 0.06), more likely to have ulcerative colitis (58.0% vs. 44.4%, *p* < 0.001) and comorbidities (48.9% vs. 38.9%; p < 0.01), and more frequently residing in Northern Italy (73.1% vs. 46.0%, *p* < 0.001) than patients of the second wave. There were no significant differences between pandemic waves in sex (male: 54.3% vs. 53.3%, *p* = 0.82) or frequency of active IBD (44.3% vs. 39.0%, *p* = 0.18). The rates of negative outcomes were significantly higher in the first than second wave: pneumonia (27.8% vs. 11.7%, *p* < 0.001), hospital admission (27.4% vs. 9.7%, *p* < 0.001), ventilatory support (11.9% vs. 5.4%, *p* < 0.003) and death (5.5% vs. 1.8%, *p* < 0.007).

**Conclusion:**

Between the first and second SARS-CoV-2 pandemic waves, demographic, clinical and geographical features of IBD patients were different as were the symptoms and outcomes of infection. These differences are likely due to the different epidemiological situations and diagnostic possibilities between the two waves.

**Supplementary Information:**

The online version contains supplementary material available at 10.1186/s12876-023-02841-0.

## Background

Severe acute respiratory syndrome coronavirus 2 (SARS-CoV-2) is a new member of the coronavirus family, first identified in China at the end of 2019. In the beginning of 2020, it started spreading worldwide, leading the World Health Organization in March 2020 to declare a pandemic of coronavirus disease 2019 (COVID-19) [1]. The clinical picture of COVID-19 is extremely heterogeneous, ranging from mild disease to pneumonia and even acute respiratory distress syndrome, which is potentially fatal [2].

After China, Europe was the first region of the world to be affected by the pandemic. In particular, in Italy the spread of infection started in February 2020 and, from then until 31 December 2020, COVID-19 caused 10% of all deaths, and 14% of all deaths in Northern Italy, 7% in Central Italy, and 5% in Southern Italy [3, 4]. A first pandemic "wave" lasted from February to May 2020, when the infection spread rapidly, especially in the north, causing a very high number of deaths and placing huge stress on the health care system, leading to the lockdown. In September 2020, a second pandemic wave started when new cases increased exponentially for several weeks and containment measures were implemented at regional and national levels [3]. During this second wave, the impact of COVID-19 on the different macroregions of Italy was homogeneous [5].

In Italy, differences in geographical impact were not the only difference between the first and second waves. Patients affected in the second wave were younger, had fewer comorbidities, and experienced milder symptoms, with overall better short-term outcome and lower mortality (43.3% in first wave vs. 11.5% in second wave) [6]. These differences were also observed in other countries [7–12].

In patients with inflammatory bowel disease (IBD), the risk of SARS-CoV-2 infection is not higher than among the general population [13]. Furthermore, the risk of a severe COVID-19 course, requiring ventilation or leading to death, is similar in IBD patients to that of the general population [14]***.*** Also, the main risk factors for negative COVID-19 outcomes are similar to those in the general population, and are older age [13, 15–20], male sex [19], and comorbidities [13, 15–19, 21]. Risk factors for negative COVID-19 outcomes related to IBD are active disease [14, 15, 22] and ulcerative colitis [15]. The precise impact of medication on outcome is not fully defined, but so far no therapy has been shown to negatively affect COVID-19 course [16, 20, 22]. Despite these studies on COVID-19 in IBD patients, no study has yet compared the impact of the infection between first and second pandemic waves. Therefore, we assessed epidemiology and clinical outcomes of SARS-CoV-2 infection in the IBD population in Italy between the first and second pandemic waves.

## Methods

### Patients

This observational, longitudinal, multicentre study was supported and coordinated by the Italian Group for the Study of Inflammatory Bowel Disease (IG-IBD). The study protocol was approved by the ethics committees of participating IBD centres from across Italy. Patients at participating IBD centres were consecutively included in the study if they had an established diagnosis of Crohn’s disease (CD) or ulcerative colitis (UC) for at least 6 months and a SARS-CoV-2 infection, defined as the polymerase chain reaction (PCR)-confirmed presence of SARS-CoV-2 genome in a nasopharyngeal swab. Patients were enrolled in two periods: (i) first wave, from 1 March 2020 to 31 May 2020; and (ii) second wave, from 15 September to 15 December 2020.

The following data were collected for each patient at enrollment: sex, age, comorbidities, macroregion of residence (Northern Italy, Central Italy, and Southern Italy including the islands), type of IBD (UC or CD), year of IBD diagnosis, IBD activity, and ongoing IBD therapy. IBD activity was measured according to the partial Mayo score for UC patients [23] and the Harvey-Bradshaw index (HBI) for CD patients [24].

Data on COVID-19 symptoms and course were collected for each patient. COVID-19 symptoms were classified as respiratory (pharyngodynia, cough, rhinitis, dyspnoea), gastrointestinal (diarrhoea, nausea, vomiting or abdominal pain), systemic (fever, arthralgia, myalgia, fatigue or anorexia), or dysgeusia or anosmia. COVID-19 outcome was considered negative if there was at least one of the following conditions: pneumonia, need for hospital admission, need for ventilatory support, or death. A severe negative outcome was defined as the need for ventilatory support (continuous positive airway pressure, noninvasive mechanical ventilation or intubation) or death.

### Statistical analysis

Continuous normal variables were compared with Student's *t* test. Fisher’s exact test and chi-square test were used for categorical variables. Statistical significance was set at a *p* value of 0.05 or less. Statistical analyses were done using MedCalc software (v.18.9.1; MedCalc Software, Ostend, Belgium).

## Results

Overall, 937 IBD patients were enrolled, including 219 in the first wave and 718 in the second wave (Table 1). Patients in the first wave were older than those in the second wave (mean, 46.3 vs. 44.1 years, *p* = 0.06). Moreover, there was a higher prevalence of UC patients in the first than second wave (58.0% vs. 44.4%; *p* < 0.01) and a higher percentage of patients with comorbidities (48.9% vs. 38.9%; *p* < 0.01). No significant difference was found in the prevalence of patients with active disease. Furthermore, no significant differences were found in the frequency of use of different therapies (Table 2). In terms of geographical distribution, the percentage of patients residing in Northern Italy was significantly higher in the first than second wave (73.1% vs. 46.0%; *p* < 0.01).

**Table 1:** Demographic and clinical characteristics of IBD patients during first and second waves of the COVID-19 pandemic in Italy

Characteristic	First wave (*n* = 219)	Second wave (*n* = 718)	*p* value^a^
Male sex, n (%)	119 (54.3)	383 (53.3)	0.82
Age, years, mean (SD)	46.3 (16.2)	44.1 (15.4)	0.06
Ulcerative colitis, n (%)	127 (58.0)	319 (44.4)	< 0.01
Disease duration, years, mean (SD)	13.7 (9.6)	13.1 (10.0)	0.45
Active disease, n (%)	97 (44.3)	280 (39.0)	0.18
Comorbidities, n (%)	107 (48.9)	279 (38.9)	< 0.01
Smoking habit, n (%)	37 (16.9)	111 (15.5)	0.60
Macroregion of residence, n (%)			< 0.001
Northern Italy	160 (73.1)	330 (46.0)	
Central Italy	48 (21.9)	204 (28.4)	
Southern Italy	11 (5.0)	184 (25.6)	

^a^Student's* t* test for age and disease duration; Chi-square test and Fisher's exact test for the other variables

**Table 2:** Ongoing therapy in IBD patients with a SARS-CoV-2 infection, by pandemic wave

**IBD therapy, n (%)**	**First wave (*n* = 219)**	**Second wave (*n* = 718)**	***p* value**^a^
None	17 (7.8)	39 (5.4)	0.20
Salicylates	111 (50.7)	381 (53.1)	0.58
Steroids	22 (10.0)	100 (13.9)	0.17
Immunosuppressors	22 (10.0)	79 (11.0)	0.80
Immunosuppressors + anti-TNF agents	6 (2.7)	16 (2.2)	0.62
Anti-TNF agents	76 (34.7)	270 (37.6)	0.47
Vedolizumab	33 (15.1)	82 (11.4)	0.16
Ustekinumab	10 (4.6)	41 (5.7)	0.61
Other^b^	4 (1.8)	9 (1.3)	0.51

*TNF* Tumour necrosis factor

^a^Fisher's exact test

^b^Including risankizumab, ozanimod, filgotinib, and apremilast

Symptoms related to SARS-CoV-2 infection are shown in Table 3. The percentage of asymptomatic patients was significantly lower during the first than second wave (9.1% vs. 15.6%, *p* < 0.01). In contrast, respiratory (68.9% vs. 53.9%) and systemic (78.5% vs. 66.7%) symptoms were significantly more frequent in the first wave (*p* < 0.01 for both). No significant differences were found for the other symptoms.

**Table 3:** SARS-CoV-2 infection-related symptoms among IBD patients, by pandemic wave

**Symptoms, n (%)**	**First wave (*n* = 219)**	**Second wave (*n* = 718)**	***p* value**^a^
None	20 (9.1)	112 (15.6)	0.01
Respiratory	151 (68.9)	387 (53.9)	< 0.01
Gastrointestinal	57 (26.0)	142 (19.8)	0.06
Systemic	172 (78.5)	479 (66.7)	< 0.01
Dysgeusia or anosmia	75 (34.2)	251 (35.0)	0.87

^a^Fisher's exact test

**Fig. 1:**
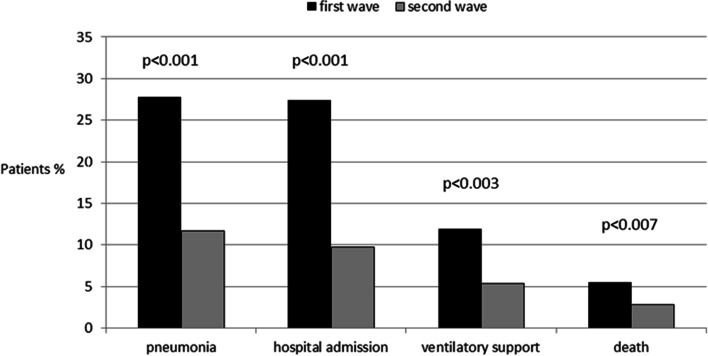
Negative COVID-19 outcomes in IBD patients, by pandemic wave

Overall, 145 patients had a negative outcome: 61 in the first wave, and 84 in the second wave. The rates of individual negative outcomes were all significantly higher in the first than second wave: pneumonia (27.8% vs. 11.7%, *p* < 0.001), hospital admission (27.4% vs. 9.7%, *p* < 0.001), ventilatory support (11.9% vs. 5.4%, *p* < 0.003) and death (5.5% vs. 1.8%, *p* < 0.007) (Fig. 1).

Among patients with negative outcomes, there were no significant differences between the first and second waves in terms of mean age, presence of comorbidities and rate of active disease (Table 4). Instead, a significant difference was observed regarding the geographical distribution, with more patients from Northern Italy in the first than second wave. Similar findings were observed limiting the analysis to patients with severe COVID-19 (Supplementary Table 1).

**Table 4:** Demographic and clinical characteristics of IBD patients with negative COVID-19 outcomes, by pandemic wave

**Characteristic**	**First wave (*n* = 61)**	**Second wave (*n* = 84)**	***p* value**^a^
Age, years, mean (SD)	55.4 (15.9)	58.1 (15.1)	0.28
Comorbidities, n (%)	41 (67.2)	61 (72.6)	0.58
Active disease, n (%)	30 (49.1)	47 (55.9)	0.50
Macroregion of residence, n (%)			< 0.002
Northern Italy	51 (83.6)	46 (54.8)	
Central Italy	7 (11.5)	22 (26.2)	
Southern Ital*y*	3 (4.9)	16 (19.0)	

^a^Student's* t* test for age; Fisher's exact test

## Discussion

This study compared demographic and clinical features of IBD patients with a SARS-CoV-2 infection between the first and second pandemic waves in Italy. During the first wave, 73.1% of SARS-CoV-2-infected IBD patients were residents of Northern Italy, while during the second wave this percentage was less (46.0%). Moreover, in the first wave, patients were older (*p* = 0.06) and significantly more likely to have UC and comorbidities than patients in the second wave. In contrast, no differences were found between pandemic waves in the frequency of active disease or use of different IBD therapies. Regarding the clinical presentation of SARS-CoV-2 infection, more patients were asymptomatic in the second than first wave; systemic and respiratory symptoms were significantly less frequent than in the first wave. Gastrointestinal symptoms were slightly more frequent in the first pandemic wave and occurred at a percentage lower than that reported by other authors [25]. Negative outcomes (pneumonia, hospital admission, need for ventilatory support, or death) were significantly more frequent in the first than second wave and in patients residing in Northern Italy.

Our findings in IBD patients are consistent with the general epidemiological situation in Italy in the two pandemic waves [5, 6, 25]. In particular, the different geographical distributions and rates of negative outcomes in IBD patients with a SARS-CoV-2 infection in the two waves reflect those of the general Italian population [5]. The higher rate of negative COVID-19 outcomes during the first pandemic wave is likely due to the fact that diagnosed patients in Italy had more severe disease and were more often symptomatic. These differences are related to differences in how the national health care system organized the screening for and treatment of SARS-CoV-2 infections. During the first wave, only patients admitted to hospital or with moderate-to-severe symptoms underwent molecular testing, whereas asymptomatic subjects and patients with mild symptoms were not tested (and only required to self-isolate). The limited testing in the first wave contributed to an underestimation of the total number of cases and consequentially increased the percentages of patients with severe and fatal COVID-19. On the other hand, during the second wave, there was better adherence to containment measures such as social distancing, sanitizing and use of facial masks and, at the same time, greater availability of testing [26]. As shown by Dorrucci et al. [27], the excess death rate for all of Italy was similar between the two waves (31% during the first and 35% during the second wave). Moreover, the overall estimated mortality in Italian Internal Medicine wards was also similar in the two pandemic waves [28]. On the other hand, the excess death rate in Northern Italy reduced from 60% in the first wave to 42% in the second wave, while in the rest of Italy it increased [27]. Thus, the risks of SARS-CoV-2 infection and of COVID-19 in IBD patients may be similar to those in the general population. These observations also suggest that the national lockdown during the first wave was effective in preventing the virus from spreading outside of Northern Italy.

Our findings that the incidence of SARS-COV-2 infection and negative outcomes (in the two pandemic waves) paralleled what was observed in the general population are coherent with what was reported globally for IBD patients [29]. A similar lack of difference in negative outcomes of SARS-CoV-2 infection between the two pandemic waves was observed in patients with cirrhosis [30].

This study reports findings from a large cohort of consecutive IBD patients with COVID-19 from a single country, where the overall management of COVID-19 has been relatively homogeneous in the two pandemic waves. The main limitations of our study are represented by that the results may not be applicable in settings that differ in terms of epidemiological course as well as social, cultural, political and economic backgrounds and by the lack of some additional information about patients’ characteristics (namely Body Mass Index) and of a control group (e.g. general population).

## Conclusions

Demographic, clinical and geographical features of IBD patients with SARS-CoV-2 infection were different between first and second waves. Moreover, symptoms and outcomes of SARS-CoV-2 infection were different between the two pandemic waves. These findings are consistent with those observed in the general population and are likely due to the different epidemiological situations and diagnostic possibilities between the two periods. Our findings reinforce the message that IBD is not a risk factor for worse outcomes of SARS-CoV-2 infection.

## Supplementary Information

**Additional file 1:** **Supplementary Table 1.** Clinical characteristics of IBD patients with severe COVID-19 outcomes,by pandemic wave

## Data Availability

The datasets used and/or analysed during the current study available from the corresponding author on reasonable request.

## Abbreviations

COVID-19: Coronavirus disease 2019
IBD: Inflammatory Bowel Disease
HBI: Harvey-Bradshaw index
UC: Ulcerative Colitis
CD: Crohn’s disease

## References

[1] https://www.who.int/director-general/speeches/detail/who-director-general-s-opening-remarks-at-the-media-briefing-on-covid-19---11-march-2020 n.d.

[2] Rodriguez-Morales AJ, Cardona-Ospina JA, Gutiérrez-Ocampo E, Villamizar-Peña R, Holguin-Rivera Y, Escalera-Antezana JP (2020). Clinical, laboratory and imaging features of COVID-19: a systematic review and meta-analysis. Travel Med Infect Dis.

[3] https://www.istat.it/it/files/2021/03/Report_ISS_Istat_2020_5_marzo.pdf n.d.

[4] Michelozzi P, de’Donato F, Scortichini M, de Sario M, Noccioli F, Rossi P (2020). Mortality impacts of the coronavirus disease (COVID-19) outbreak by sex and age: rapid mortality surveillance system, Italy, 1 February to 18 April 2020. Eurosurveillance.

[5] ISTAT. Impatto dell’epidemia COVID-19 sulla mortalità totale della popolazione residente primo quadrimestre 2020. 2020.

[6] Portacci A, Carpagnano GE, Tummolo MG, Santomasi C, Palma L, Fasano D (2021). COVID-19 clinical phenotypes and short-term outcomes: differences between the first and the second wave of pandemic in Italy. Expert Rev Respir Med.

[7] Hoogenboom WS, Pham A, Anand H, Fleysher R, Buczek A, Soby S (2021). Clinical characteristics of the first and second COVID-19 waves in the Bronx, New York: a retrospective cohort study. Lancet Reg Heal Am.

[8] Karagodin I, Singulane CC, Besser SA, Singh A, Addetia K, DeCara JM (2021). Comparison of clinical and echocardiographic features of first and second waves of COVID-19 at a large, tertiary medical center serving a predominantly African American patient population. Int J Cardiovasc Imaging.

[9] Siegfried S, Bopp M, Günthard H, Keiser O, Weibull CE, Crowther M (2021). Original research: assessing relative COVID-19 mortality during the second wave: a prospective Swiss population-based study. BMJ Open.

[10] Fan G, Yang Z, Lin Q, Zhao S, Yang L, He D (2021). Decreased case fatality rate of COVID-19 in the second wave: a study in 53 countries or regions. Transbound Emerg Dis.

[11] Soriano V, Ganado-Pinilla P, Sanchez-Santos M, Gómez-Gallego F, Barreiro P, de Mendoza C (2021). Main differences between the first and second waves of COVID-19 in Madrid. Spain Int J Infect Dis.

[12] Iftimie S, Lopez-Azcona AF, Vallverdu I, Hernandez-Flix S, De Febrer G, Parra S (2021). First and second waves of coronavirus disease-19: a comparative study in hospitalized patients in Reus, Spain. PLoS One.

[13] Attauabi M, Poulsen A, Theede K, Pedersen N, Larsen L, Jess T (2021). Prevalence and outcomes of COVID-19 among patients with inflammatory bowel disease-a danish prospective population-based cohort study. J Crohns Colitis.

[14] Singh S, Khan A, Chowdhry M, Bilal M, Kochhar GS, Clarke K (2020). Risk of severe coronavirus disease 2019 in patients with inflammatory bowel disease in the United States: a multicenter research network study. Gastroenterology.

[15] Bezzio C, Saibeni S, Variola A, Allocca M, Massari A, Gerardi V (2020). Outcomes of COVID-19 in 79 patients with IBD in Italy: an IG-IBD study. Gut.

[16] Brenner EJ, Ungaro RC, Gearry RB, Kaplan GG, Kissous-Hunt M, Lewis JD (2020). Corticosteroids, but not tnf antagonists, are associated with adverse COVID-19 Outcomes in patients with inflammatory bowel diseases: results from an international registry. Gastroenterology.

[17] Singh AK, Jena A, Kumar-M P, Sharma V, Sebastian S (2021). Risk and outcomes of coronavirus disease in patients with inflammatory bowel disease: a systematic review and meta-analysis. United Eur Gastroenterol J.

[18] Lamb CA, Sebastian S, Kent AJ, Segal JP, Gonzalez HA, Brookes MJ (2021). Letter: risk of severe COVID-19 outcomes associated with inflammatory bowel disease medications—reassuring insights from the United Kingdom PREPARE-IBD multicentre cohort study. Aliment Pharmacol Ther.

[19] Meyer A, Semenzato L, Zureik M, Weill A, Carbonnel F, Dray-Spira R (2021). Risk of severe COVID-19 in patients treated with IBD medications: a French nationwide study. Aliment Pharmacol Ther.

[20] Bezzio C, Armuzzi A, Furfaro F, Ardizzone S, Milla M, Carparelli S (2021). Therapies for inflammatory bowel disease do not pose additional risks for adverse outcomes of SARS-CoV-2 infection: an IG-IBD study. Aliment Pharmacol Ther.

[21] Derikx LAAP, Lantinga MA, De Jong DJ, Van Dop WA, Creemers RH, Römkens TEH (2021). Clinical outcomes of Covid-19 in patients with inflammatory bowel disease: a nationwide cohort study. J Crohns Colitis.

[22] Lukin DJ, Kumar A, Hajifathalian K, Sharaiha RZ, Scherl EJ, Longman RS (2020). Baseline disease activity and steroid therapy stratify risk of COVID-19 in patients with inflammatory bowel disease. Gastroenterology.

[23] Schroeder KW, Tremaine WJ, Ilstrup DM (1987). Coated oral 5-aminosalicylic acid therapy for mildly to moderately active ulcerative colitis. a randomized study. N Engl J Med.

[24] Harvey RF, Bradshaw JM (1980). A simple index of Crohn’s-disease activity. Lancet (London, England).

[25] Viganò C, Massironi S, Pirola L, Cristoferi L, Fichera M, Bravo M (2020). COVID-19 in patients with inflammatory bowel disease: a single-center observational study in Northern Italy. Inflamm Bowel Dis.

[26] Bongiovanni M, Arienti R, Bini F, Bodini BD, Corbetta E, Gianturco L (2021). Differences between the waves in Northern Italy: how the characteristics and the outcome of COVID-19 infected patients admitted to the emergency room have changed. J Infect.

[27] Scoppetta C, Casciato S, Di Gennaro G (2021). Lethality rate of the two waves of the COVID-19 pandemic in Italy. Eur Rev Med Pharmacol Sci.

[28] Dorrucci M, Minelli G, Boros S, Manno V, Prati S, Battaglini M (2021). Excess mortality in Italy during the COVID-19 pandemic: assessing the differences between the first and the second wave, year 2020. Front Public Heal.

[29] Kaplan GG, Underwood FE, Coward S, Agrawal M, Ungaro RC, Brenner EJ (2022). The multiple waves of COVID-19 in patients with inflammatory bowel disease: a temporal trend analysis. Inflamm Bowel Dis.

[30] Elhence A, Vaishnav M, Biswas S, Anand A, Gunjan D, Kedia S (2022). Predictors of in-hospital outcomes in patients with cirrhosis and coronavirus Disease-2019. J Clin Exp Hepatol.

