# Supplementary material for: SARS-CoV-2 infection in patients with inflammatory bowel disease: comparison between the first and second pandemic waves
Source: BMC Gastroenterol. 2023 Jul 5;23:230. doi: 10.1186/s12876-023-02841-0 (PMC10324268; doi:10.1186/s12876-023-02841-0)
Supplement: Supplementary file 1 — Additional file 1: Supplementary Table 1. Clinical characteristics of IBD patients with severe COVID-19 outcomes,by pandemic wave [file 12876_2023_2841_MOESM1_ESM.docx]

**Supplementary Table 1** Clinical characteristics of IBD patients with severe COVID-19 outcomes, by pandemic wave

| **Characteristic** | | **First wave**  **(n = 31)** | **Second wave**  **(n = 44)** | **p-value**^a^ |
| --- | --- | --- | --- | --- |
| Age, years, mean (SD) | | 59.3 (13.5) | 60.5 (13.0) | 0.69 |
| Comorbidities, n (%) | | 21 (67.7) | 33 (75.0) | 0.69 |
| Active disease, n (%) | | 15 (48.4) | 25 (56.8) | 0.49 |
| Ongoing therapy, n (%) | |  |  |  |
|  | None | 1 (3.2) | 3 (6.8) | 0.64 |
|  | Salicylates | 25 (80.6) | 33 (75.0) | 0.78 |
|  | Salicylates + anti-TNF agents | 3 (9.7) | 3 (6.8) | 0.69 |
|  | Steroids | 6 (19.3) | 9 (20.4) | 1.00 |
|  | Immunosuppressors | 2 (6.5) | 5 (11.4) | 0.69 |
|  | Immunosuppressors + anti-TNF agents | 0 (0) | 0 (0) | - |
|  | Anti-TNF agents | 5 (16.1) | 6 (13.6) | 0.75 |
|  | Vedolizumab | 5 (16.1) | 8 (18.2) | 1.00 |
|  | Ustekinumab | 0 (0) | 0 (0) | - |
|  | Other^b^ | 0 (0) | 0 (0) | - |

^a^ Student's *t* test for age and disease duration; Fisher's exact test for the other variables.

^b^ Including risankizumab, ozanimod, filgotinib, and apremilast.

TNF, tumour necrosis factor.
